# Genome Announcement: The Draft Genome of the Carrot Cyst Nematode *Heterodera Carotae*

**DOI:** 10.2478/jofnem-2022-0014

**Published:** 2022-05-29

**Authors:** Catherine L. Wram, Cedar N. Hesse, Zafar A. Handoo, F. Hugo Pacheco, Inga A. Zasada

**Affiliations:** 1 USDA-ARS Horticultural Crops Research Unit 3420 NW Orchard Ave, Corvallis, OR 97331; 2 USDA-ARS Mycology and Nematology Genetic Diversity and Biology Laboratory, Beltsville, MD 20705; 3 Servicio Agrícola y Ganadero (SAG), División de Protección Agrícola y Forestal y Red SAG de Laboratorios, Santiago, Chile

**Keywords:** carrot cyst nematode, draft genome, genomics, *Heterodera carotae*

## Abstract

*Heterodera carotae*, the carrot cyst nematode, is a significant pest affecting carrot globally. Here we present the draft genome of *H. carotae*, which was generated from short read libraries from Illumina HiSeq technology, and the corresponding genome annotation.

The carrot cyst nematode, *Heterodera carotae*, is a considerable pest affecting carrot growing regions around the world, including much of Europe, South Africa, Mexico, Cyprus, Chile, Ontario, Canada, and Michigan in the United States (Berney and Bird, 1992; Subbotin *et al*., 2010; Yu *et al*., 2017; Escobar-Avila *et al*., 2018; Madani *et al*., 2018; CABI, 2021). Although *H. carotae* has a host range limited to species in the Apiaceae family, mainly wild and cultivated carrot (*Daucus carota*), infestation of commercial production fields by this nematode can be devastating with yield losses between 12% and 80% (Greco *et al*., 1993; Subbotin *et al*., 2010). Despite the significance of this pest, there are no genomic or transcriptomic resources publically available for this nematode. To date, research utilizing molecular tools in *H. carotae* has been limited to the development of a molecular diagnostic for populations of *H. carotae* from Ontario, Canada, and Northern Italy (Madani *et al*., 2018) and microsatellite genotyping of *H. carotae* to examine the gene flow in French populations (Esquibet *et al*., 2020). The *H. carotae* genome will provide a valuable resource to researchers tackling pest management of *H. carotae* and to the wider cyst nematode research community.

In 2019, 1 kg of soil was collected from a carrot field in Calama, Antofagasta region, Chile. The soil was placed in a paper bag. At the Nematology Laboratory of Agriculture and Livestock Service (Santiago, Chile) the soil samples were placed on trays and allowed to dry for at least a week. Cysts were then extracted from 250 g of soil using a modified Fenwick can (Fenwick, 1940). The cysts were picked from the samples and placed in DESS (Yoder *et al*., 2006). The samples were shipped to the USDA-ARS Horticultural Crops Research Unit in Corvallis, OR for DNA extraction and sequencing. From the picked cysts, 25 were hand selected, broken open in sterilized water using a scalpel, and the eggs were collected using a Pasteur pipette. Genomic DNA was extracted from the eggs using the QIAmp DNA Micro Kit (Qiagen, Hilden, Germany) following manufacturer's instructions. Sequencing and library preparation were done at the Center for Qualitative Life Sciences at Oregon State University (Corvallis, OR). The NEBNext Ultra II DNA Library Prep Kit for Illumina (San Diego, CA) was used to prepare a whole genome shotgun library from egg DNA and sequencing was performed on the Illumina HiSeq 3000 platform. The cysts were also sent to the USDA-ARS Mycology and Nematology Genetic Diversity and Biology Laboratory (Beltsville, MD) to confirm species identification through morphometrics.

To assemble the genome, raw sequencing data was first subjected to adaptor removal and quality control (*Q* = 20) using Trim Galore! v. 0.6.6 (Krueger, 2020) resulting in 33,127,853 150-bp paired-end reads. Using the filtered reads, a de novo assembly was generated using metaSPAdes v. 3.15.3 (Nurk *et al*., 2017). The de novo meta-assembly was visualized using the Blob Tools workflow (Kumar *et al*., 2013) to identify and remove contaminating contigs. Each contig in the meta-assembly was assigned a phylum ID based on BLAST similarity (*E*-value < 10e^−25^) to sequences in the NCBI “nt” database or other plant-parasitic nematode genomes (*H. schatti* [NCBI BioProject:PRJNA722882], *H. glycines* [NCBI BioProject:PRJNA381081], *Meloidogyne incognita* [ENA Project:PRJEB8714], *Radopholus similis* [NCBI BioProject:PRJNA541590], *Globodera rostochiensis* [ENA Project: PRJEB13504], *Globodera pallida* [ENA Project: PRJEB123]). The read coverage and GC content of each contig were used to visualize the assembly. Reads that belonged to contigs that were identified as Nematoda or had no assigned identity were retained and used to assemble the *H. carotae* genome using de novo assembler SPAdes v. 3.15.3 (Bankevich *et al*., 2012). This second assembly was again subjected to the Blob Tools BLAST-based filtering of reads to remove any remaining contamination before reassembly using SPAdes to achieve the final assembly of the *H. carotae* genome. The final assembly was visualized using each contig's coverage, GC content, and phylum identity (Supplementary Fig. 1). Using Pilon v. 1.22 (Walker *et al*., 2014) gap-filling, mis-assembly correction, base correction, and scaffolding were performed on the final assembly. The genome statistics of the corrected version of the final assembly were calculated using QUAST (Gurevich *et al*., 2013).

The goal of this genome sequencing effort was to rapidly provide usable data that was of sufficient quality to the nematology community to expand genomic resources available for cyst nematodes. The scaffolded assembly of the *H. carotae* genome was 95,118,078 bp in 17,839 scaffolds (Table 1). Two other *Heterodera* species have publicly available genomes, *H. glycines* and *H. schachtii*, which are 1.66 and 1.88X larger than the *H. carotae* genome, respectively (Table 1). Unlike *H. carotae*, the genomes for *H. glycines* and *H. schachtii* were assembled in fewer segments and consisted of 9 and 395 scaffolds, respectively. Using the raw data as input and a kmer size of 21, GenomeScope (Vurture *et al*., 2017) was used to determine the potential genome size and repeat length for the *H. carotae* genome which were estimated at 120 Mb and 81 Mb, respectively. The level of duplication and the number of repetitive regions is unclear in the *H. schachtii* genome. The *H. glycines* genome contains 34% repeated regions and 18.7 Mb of tandem duplicates (Masonbrink *et al*., 2019), indicating that the fragmented assembly of *H. carotae* may be due in part to the inability of Illumina sequencing to resolve repetitive and low complexity portions of the genome. Additionally, some repetitive regions of the genome may have been lost due to the high stringency standards used when BLAST filtering for contamination. The N50 for *H. glycines* and *H. schachtii* are 17 Mb and 1.2 Mb, respectively, whereas the N50 for *H. carotae* is 13,935 bp (Table 1). The GC content of each genome is relatively similar with 39.39%, 36.66%, and 33.23% for *H. carotae*, *H. glycines*, and *H. schachtii,* respectively (Table 1).

**Table 1:** Comparison of the genome assembly statistic of *Heterodera* species.

**Assembly statistic**	***H. glycines* (PRJNA381081)^a^**	***H. schachtii* (PRJNA722882)^a^**	***H. carotae* (PRJNA774818)^a^**
Size (bp)	157,978,452	179,246,932	95,118,078
Number of scaffolds	9	395	17,839
Largest scaffold (bp)	23,985,585	6,046,013	113,425
GC content (%)	36.66	33.23	39.39
N50 value (bp)	17,907,690	1,273,070	13,935
No. contigs >5,000 bp	9	395	5,030
No. contigs >10,000 bp	9	359	2,755
No. contigs >25,000 bp	9	309	699
No. contigs >50,000 bp	9	269	103

Complete BUSCOs (%)	1,400 (44.7)	1,422 (45.4)	1,259 (40.2)
Complete and single-copy BUSCOs (%)	1,291 (41.2)	1,372 (43.8)	1,238 (39.5)
Complete and duplicated BUSCOs (%)	109 (3.5)	50 (1.6)	21 (0.7)
Fragmented BUSCOs (%)	109 (3.5)	120 (3.8)	143 (4.6)
Missing BUSCOs (%)	1,622 (51.8)	1,589 (50.8)	1,729 (55.2)

Predicted protein coding genes	29,679	26,768	17,212

a NCBI GenBank BioProject accession number.

To determine the completeness of the *H. carotae* genome BUSCO v.5.2.2 was used (Simão *et al*., 2015). BUSCO was also run on the *H. glycines* and *H. schachtii* genomes for comparison (Table 1). The *H. carotae* genome was ~5% less complete than the *H. glycines* and *H. schachtii* genomes. All three genomes shared 927 complete genes and 1,391 missing BUSCO genes. Across all three genomes, there are 1,613 complete BUSCO genes. In Supplementary Fig. 2, a Venn diagram can be found depicting the overlap of complete BUSCO genes across the three *Heterodera* species. Although all three genomes had complete BUSCO scores below 50% they are in line with other plant-parasitic nematode genomes that range in completeness from 40% to 60% (Howe *et al*., 2016, 2017).

In addition to confirming specimen identity using morphometrics, *coxI* and *hsp90* were used to place the specimen in a phylogenetic context. All available sequences of *coxI* for *H. carotae* and *Heterodera cruciferae* were obtained from NCBI, along with a random selection of *coxI* accessions from 31 other *Heterodera* species, and five accessions of *coxI* from *Meloidogyne* species as an outgroup. All NCBI accessions used are denoted in Supplementary Fig. 3. The *coxI* sequence was retrieved from the *H. carotae* assembly using usearch11 (Edgar, 2010) with *H. carotae* isolate U45 (NCBI Accession KX463301.1) as the search sequence. All sequences were aligned using MUSCLE v.3.8.31 (Edgar, 2004), Newick phylogenetic trees were created using FastTree v.2.1.10 with default parameters (Price *et al*., 2010), and trees were visualized using TreeGraph 2 (Stöver and Müller, 2010). As shown in Supplementary Fig. 3, *H. carotae* from this study formed a clade with three other *H. carotae* isolates collected from carrot in the Tepeaca Valley, Puebla, Mexico (Escobar-Avila *et al*., 2018) supported by a 0.836 bootstrap value.

The same process described above was used to examine the phylogenetics of *hsp90* from the Chilean *H. carotae. H. carotae* clone 2449 (NCBI Accession JQ316187.1) was used as the search sequence to retrieve *hsp90* from the *H. carotae* genome. *Hsp90* sequences were obtained from NCBI for phylogenetic comparison to the *H. carotae* in this study from four *Globodera* species, six *Heterodera* species, all available *H. carotae* accessions, two *Cactodera* species, and six *Meloidogyne* species as an outgroup. NCBI accessions and species are denoted in Supplementary Fig. 4. Similar to the *coxI* results, the *H. carotae* from Chile fell into a clade separate from other *Heterodera* species but with other *H. carotae* isolates, supported by a bootstrap value of 0.943 (Supplementary Fig. 4).

The *H. carotae* genome was annotated using BRAKER v. 2.1.5 (Hoff *et al*., 2016) with protein hints from *H. glycines* (Masonbrink *et al*., 2019). In order to compare across other *Heterodera* species genomes, *H. schachtii* was also annotated with the same parameters as *H. carotae*. *H. schachtii* and *H. glycines* have 9,556 and 12,467 more genes, respectively, than *H. carotae,* which has 17,212 protein coding genes identified in the genome (Table 1). In the *H. glycines* genome there is a strong colocalization of effector genes in highly duplicated scaffolds (Masonbrink *et al*., 2019). Some protein coding genes could be lost in *H. carotae* genome due to the collapse of repeated and duplicated regions in the current assembly. Additionally, *H. glycines* was annotated with the addition of RNAseq data, which is not available for *H. carotae*. The addition of RNAseq data could both improve the predicted proteins calls in the *H. carotae* annotation and increase the number of predicted proteins.

The *H. carotae* genome assembly, annotation, and raw data can be found under the NCBI BioProject PRJNA774818. This version of the *H. carotae* genome provides a starting point for further investigation into the basic biology of *H. carotae* and a resource for the greater cyst nematode genomics community. Further sequencing and improvements to the *H. carotae* genome would increase its usefulness; however, in its current state this genome offers researchers a resource for discovering novel *H. carotae* effectors, developing diagnostic markers, or examining genomic similarities across cyst species.

## Figures and Tables

**Figure S1:**
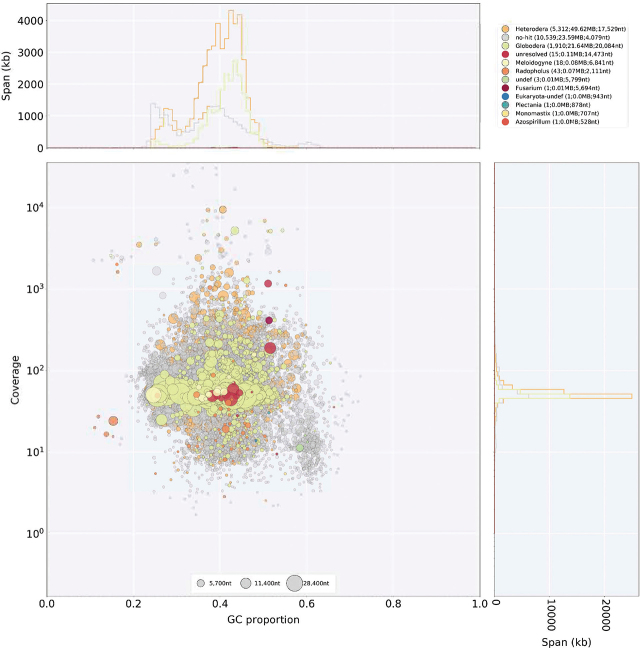
Visualization of the final *Heterodera carotae* genome assembly. Genome contigs were assembled from raw Illumina HiSeq3000 reads generated from *H. carotae* egg DNA using the assembler SPAdes. In this blobplot, assembled contigs are represented as circles with placement of circles on the *x*-axis reflecting that contig's proportion of GC bases and the position on the *y*-axis reflecting the coverage of the contig with the raw data. Contig circles are also colored based on their taxonomic identity indicated by the legend in the upper right-hand corner.

**Figure S2:**
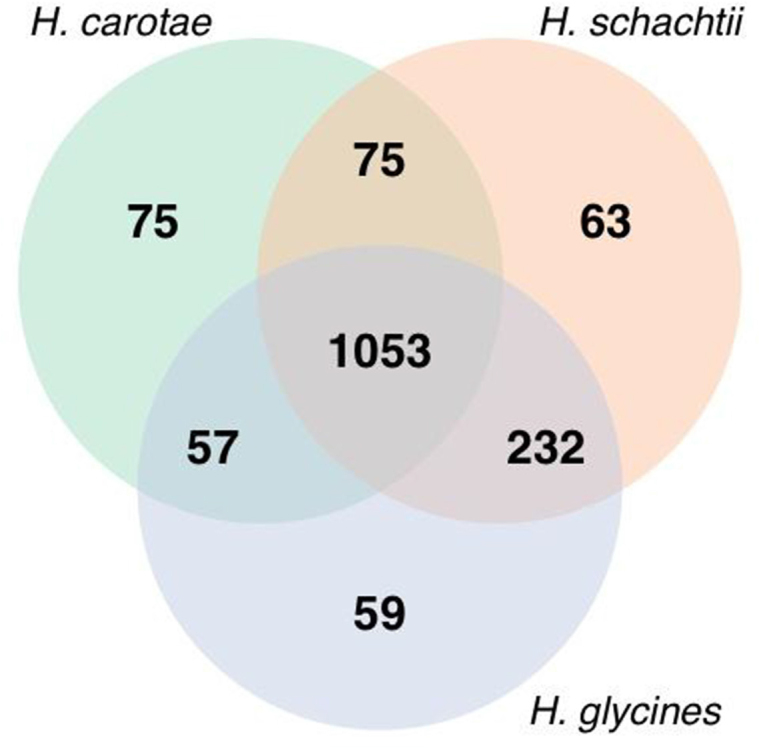
Venn diagram of complete BUSCO genes across *Heterodera glycines*, *H. carotae*, and *H. schachtii*. A BUSCO gene was counted as complete if denoted in the BUSCO analysis results as either complete or duplicated.

**Figure S3:**
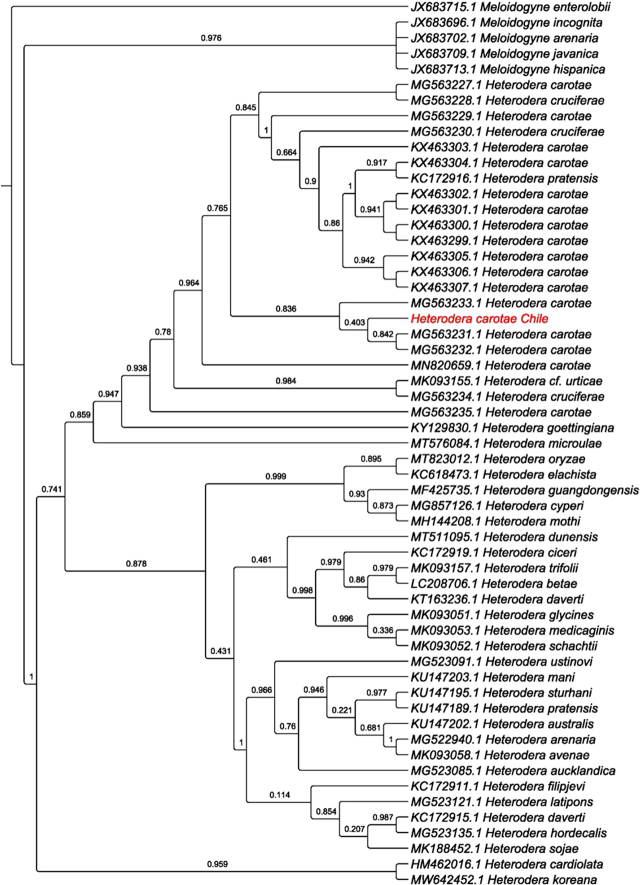
Phylogenetic tree of Chilean *Heterodera carotae* Cyclooxygenase 1 (*coxI*) gene in relation to other *Heterodera* species. The Chilean *H. carotae coxI* gene was extracted from the final assembly and aligned with all available *coxI* sequences on NCBI for H. carotae and *H. cruciferae*, a random selection of *coxI* accessions from thirty-one other *Heterodera* species, and five accessions of *coxI* from *Meloidogyne* species as an outgroup. A Newick phylogenetic tree was generated from the alignment. Bootstrap values are indicated on branch points in this tree and the placement within the tree of *H. carotae coxI* from this study highlighted in red. NCBI accessions for the sequence used are listed next to each species in the tree.

**Figure S4:**
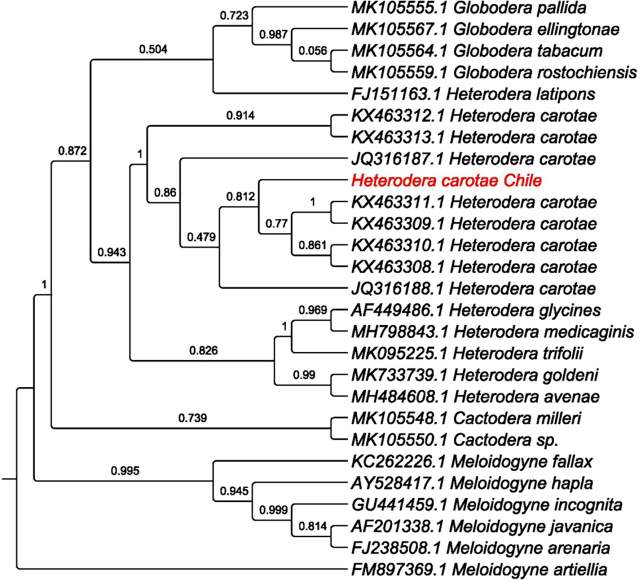
Phylogenetic tree of Chilean *Heterodera carotae* heat shock protein 90 (*hsp*90) gene in relation to other *Heterodera* species. The Chilean *H. carotae hsp90* gene was extracted from the final assembly and aligned with *hsp90* sequences from NCBI of four *Globodera* species, six *Heterodera* species, all available *H. carotae* accessions, two *Cactodera* species, and six *Meloidogyne* species as an outgroup. A Newick phylogenetic tree was generated from the alignment. Bootstrap values are indicated on branch points in this tree and the placement within the tree of *H. carotae hsp90* from this study is highlighted in red. NCBI accessions for the sequence used are listed next to each species in the tree.
